# Exploring the Bacterial Microbiome of High-Moisture Plant-Based Meat Substituted Soybean Flour with Mung Bean Protein and Duckweed Powder

**DOI:** 10.3390/biology14060735

**Published:** 2025-06-19

**Authors:** Jutamat Klinsoda, Theera Thurakit, Kullanart Tongkhao, Khemmapas Treesuwan, Kanokwan Yodin, Hataichanok Kantrong

**Affiliations:** 1 Institute of Food Research and Product Development, University of Kasetsart, Bangkok 10903, Thailand; ifrtet@ku.ac.th (T.T.); ifrkpt@ku.ac.th (K.T.); ifrkwd@ku.ac.th (K.Y.); ifrhnk@ku.ac.th (H.K.); 2 Department of Food Science and Technology, Faculty of Agro-Industry, University of Kasetsart, Bangkok 10900, Thailand; kullanart.t@ku.th

**Keywords:** microbiome, plant-based meat, mung bean protein, duckweed, alternative protein

## Abstract

(1) **Background:** This study aimed to investigate the bacterial microbiomes in the ingredients and final PBM products during a storage period of 28 days at 2–4 °C for food safety and quality. (2) **Methods:** DNA from raw ingredients (i.e., defatted soy flour, potato starch, wheat gluten, mung bean protein, and duckweed) and three PBM formulations were extracted and sequenced using 16S rRNA gene sequencing. (3) **Results:** Alpha diversity (Simpson and Shannon) was high in the raw ingredients (*p* ≤ 0.05). Beta diversity showed dissimilarities between the samples. *Firmicutes* and *Proteobacteria* were the core microflora in these ingredients. The heat-stable microbes in PBM (e.g., *Nostocaceae* in SF and *Cyanobacteriale* in MB and DW) survived after extrusion. After the ingredients were stored at room temperature, the bacterial communities shifted, with *Paucibacter* being the majority population in raw ingredients and PBM in the 2nd batch. The predictions of Potential_Pathogens related to the abundance of *Aeromonadaceae* and *Enterobacteriaceae* need to be monitored during storage. (4) **Conclusions:** Our results showed that the bacterial community in PBM containing 30% MB and 3% DW did not drastically change during 28 days of storage at cold temperatures. Uncovering bacterial microbiomes in the ingredients should be emphasized for quality and safety, as ingredients influence the microbiome in the final products.

## Simple Summary

Alternative plant-based proteins, like mung bean and duckweed, can be substituted for soybean flour to create new structural properties in producing high-moisture plant-based meat (PBM) with a moisture content of approximately 61–68%. The application of 16S rRNA gene sequencing detected major bacterial communities in the ingredients and the final PBM products. The bacterial communities in mung beans and duckweed influenced the bacterial composition of PBM. This finding demonstrates the potential risk that the survivability of spore-forming bacteria (*Bacillus*) or possible pathogens (*Enterobacteriaceae*) in the final products can compromise the safety of products when they survive the extrusion process. The data highlight the critical need for hygiene measures to investigate raw ingredients using the 16S rRNA gene, as this facilitates the exploration of major heat-stable bacteria and pathogens to improve safety and shelf-life prediction.

## 1. Introduction

The demand for plant-based diets is increasing globally. It is also proposed as an alternative to animal-based protein due to its social impact on animal ethical issues [1]. Promoting plant-based proteins over animal-based proteins improves gut health, and polyphenols in plant proteins have antioxidant properties. Recent studies have reported that plant proteins that affect glucose metabolism can reduce the risk of type 2 diabetes [2]. An increasing number of consumers have shifted towards plant-based protein (soybean) consumption and are searching for new plant protein sources such as seaweed, rapeseed, and duckweed [2,3]. Most plant-based meat analogs in the food market and from soybean flour are produced using extrusion technology. Extruders use a high-temperature, short-time (HTST) process and offer pre-cooking or pre-gelatinization of flour or starch with a significant increase in the digestibility of flour or starch. For instance, textured vegetable protein (TVP) is produced through extrusion with various pulse fibers, closely imitating the texture of animal meat [3]. Soybean isoflavone is of interest to TVP as it can reduce the risk of cancer [4]. However, the allergenicity of TVP is a significant concern for consumers with soy allergies.

Mung bean is a promising plant-based protein source for PBM due to its high protein content (25–28%) and low fat (1–2%). The isolated mung bean protein has a high-quality protein and a high amount of the amino acid profile proline, glutamic acid, arginine, leucine, and phenylalanine, which is comparable to soybean protein [1,5]. Recently, TVP was produced from mung beans via extrusion [1]; however, little information is available on their bacterial diversity, which may affect the quality of TVP products from mung beans.

Another good candidate for future food protein is duckweed, called “Wolffia.” It can be an alternative rich source of protein, replacing animal meat, and is traditionally employed as a food source in Southeast Asia, whereby *Wolffia globosa*, the most common one, is known as watermeal or Khai Nam in Thailand for cooking salads, omelets, or vegetable curries [6]. Duckweed contains 20–40% protein, 1–36% carbohydrate, and 3–7% fat per dry weight [7]. As duckweed contains high amounts of high-quality protein with a better composition of essential amino acids, it is close to WHO recommendations (4.8% Lys, 2.7% Met + Cys, and 7.7% Phe + Tyr) [7]. The fat content comprises 60% total fatty acids and 40% α-linolenic acid of fat [8], whereby carotenoids and polyphenols like flavonoids and anthocyanins are found [9]. The presence of phenolic compounds in duckweed can be a source of bioactive compounds to control and inhibit food spoilage and pathogens [10]. The water source and quality were the main factors influencing the microbial population in duckweed. The food safety quality standard for free foodborne pathogens in duckweed ingredients is not known and must be established before using duckweed in food production [6].

Advances in next-generation sequencing (NGS) have allowed us to observe a whole population of microorganisms, ingredients, and foods, including non-culturable microorganisms. The application of omics approaches can provide information on monitoring spoilage microorganisms and gain insight into the relationship between the bacterial community and product shelf life during storage [11]. For instance, Keshri et al. investigated bacterial microbiomes in sprouts in Canada for safety [12]. The food microbiome has been of increasing interest in food safety and quality for creating more sustainable food production. Food quality and safety improvements have been in global demand for food security, as recommended by government agencies [13].

Apart from the improved texture of soybean-based TVP, high-moisture PBM is an alternative product similar to the texture of meats like chicken and increases consumer acceptance [14]. Nevertheless, high moisture content (approximately 61–68%) results in the growth of microorganisms, which limits the shelf life of the products, and the limitation is that it requires frozen storage to keep products safe and extend their shelf life. Accordingly, we hypothesized that adding new protein sources with high phenolic compounds from mung beans and duckweed would change the community of microorganisms in high-moisture PBM and alter its shelf life during cold storage. Thus, this study aimed to investigate the bacterial microbiome in plant-based proteins (i.e., mung bean protein (Mb) and dried duckweed powder (Dw)) used as soy substitutes in high-moisture PBM during a storage period of 28 days at 2–4 °C and to assess changes in bacterial community between individual ingredients in high-moisture PBM, which may influence product shelf life. Insights into the major bacterial community in PBM containing mung beans and duckweed and their ingredient compositions could be used to monitor spoilage microorganisms in the raw ingredients and finished products to enhance the safety and quality of ingredients and PBM from alternative plant-based proteins.

## 2. Materials and Methods

### 2.1. Sample Preparation and Sampling

The PBM formulation included 6 raw ingredients in powder form (Sf: Defated Soy Flour, Ps: Potato Starch, Wg: Wheat Gluten, Mb: Mung bean protein (*Vigna radiata L*.), Dw: Duckweed (*Wolffia globosa*), and Rice bran oil as a texture enhancer. The protein ratios in the three PBM formulas in the experiment were modified from the formula of meat analogs reported by Prasert et al. [15]. Briefly, mung bean protein (Mb) was added to the control formula (20 MB-PBM; 20%MB *w*/*w* + 50%SF *w*/*w*) and mung bean formula (30 MB-PBM; 30%MB *w*/*w*+30%SF *w*/*w*+5%PS *w*/*w*), while the duckweed formula (DW-PBM) was prepared by 28.5%MB *w*/*w* +28.5%SF *w*/*w*+5%PS *w*/*w*+3%DW *w*/*w* (Table S1). The three PBM formulas were extruded separately, whereby the extrusion parameters for each sample were performed at approximately 130 °C of barrel temperature, with a long cooling die of 2 mm die diameter and water content of 60–70%, according to the methodology of Kantrong et al. [16] and Prasert et al. [15]. All three PBM samples, including five raw ingredients, were sampled for testing bacterial microbiomes during 28-day storage. PBM was produced in two separate batches within a 3-month interval to check alterations in the microbial communities of raw ingredients during storage at room temperature.

All three formulas of PBM were divided into two sets for the analysis of nutrient proximate compositions and shelf-life prediction by the bacterial microbiome. After production, all samples were preserved in vacuum-sealed bags and stored in a refrigeration room at 2–4 °C immediately. For the analyses of the microbiome, each package of the three formulas of PBM (20 MB-PBM, 30 MB-PBM, and DW-PBM) was sampled every 7 days during 28 days of storage (D1, D7, D14, D21, and D28) for the extraction of genomic DNA. DNA was extracted from five powdered raw ingredients (Sf, Ps, Wg, Mb, and Dw) for bacterial microbiome analysis during storage.

### 2.2. Proximate Analysis

All samples were analyzed for proximate composition, including ash, protein, fat, and moisture. The moisture content was determined using a hot air oven according to AOAC 2023a [17]. The protein content was slightly modified in the titration step by adding Boric Acid to 80 mL, as described by AOAC 2023b [18]. The fat content was examined using the Soxhlet protocol guided by AOAC 2023c [19]. The ash content in the samples was estimated using AOAC 2023d [20]. The total carbohydrate and energy contents were calculated according to the analysis method of nutrition labeling (1993) [21].

### 2.3. Bacterial Microbiome Analysis

#### 2.3.1. DNA Extraction and 16S rRNA Sequencing

Extraction of genomic DNA from three formulas of PBM (20 MB-PBM, 30 MB-PBM, and DW-PBM) and 5 raw ingredients (Sf, Ps, Wg, Mb, and Dw) was performed using Blood & Tissue kits (Qiagen, Hilden, Germany) according to the manufacturer’s instructions (Cat no./ID. 69504). The concentration and quality of the obtained DNA were measured using a Nanodrop (Thermo Fisher Scientific Inc., Waltham, MA, USA). DNA samples with A260/A280 absorbance ratios of 1.8–2.0 were submitted to Biomarker Technologies (Hong Kong) for 16S rRNA amplicon sequencing using Illumina NovaSeq 6000 (Illumina, San Diego, CA, USA).

After a quality check on gel electrophoresis, 16S rRNA sequencing targeting the V3-V4 variable regions was amplified (F:ACTCCTACGGGAGGCAGCA; R:GGACTACHVGGGTWTCTAAT) [22], and its products were purified, quantified, and homogenized to obtain a sequencing library. Library QC was performed to construct libraries according to the Biomarker Technologies (Hong Kong) protocol. Qualified libraries were sequenced using Illumina Novaseq 6000. The data files obtained by high-throughput sequencing were converted into Sequenced Reads by Base Calling analysis. The results were stored in a FASTQ format file containing the sequence information of reads and their corresponding sequencing quality information.

#### 2.3.2. Bioinformatics

For the bioinformatics analysis of the pipeline, raw reads were first filtered using Trimmomatic v0.33. The primer sequences were then identified and removed using cutadapt 1.9.1, which finally generated high-quality reads without primer sequences. De-noise was processed using the Divisive Amplicon Denoising Algorithm 2 (DADA2) in the R library, and chimeric sequences were removed, generating non-chimeric reads. Dada2 in Quantitative Insights Into Microbial Ecology 2 (QIIME2; version 2020.06) was applied to denoise sequences, generating ASVs (amplicon sequence variants). Taxonomic annotation of the sequences at the phylum, family, and genus levels was processed by Bayesian classifier using SILVA as a reference database (Version 138.2), and the common and unique features among formulas of the meat analogs at different days of storage were visualized using Venn diagrams. Alpha diversity indices (Chao1, Shannon, and Simpson) and beta diversity were calculated using the QIIME2. A Principal coordinate analysis (PCoA) plot was generated to compare the bacterial community structure based on the Bray-Curtis dissimilarity of the bacterial population among the 3 formulas of high-moisture meat analogs using R software (‘vegan’ R package; version 2025.05.0+496). Then, UPGMA clustering trees were constructed based on four distance algorithms, which showed the similarity between samples at different days of storage. Microbiome functional phenotypes were predicted for the 10 bacterial communities using the BugBase tool for functional prediction by uploading them on the web-based version of BugBase.

### 2.4. Statistical Analysis

To compare bacterial microbiomes among ingredients and three formulas of PBM and the influence of storage days, the raw read counts from each PBM sample were collapsed and normalized such that each sample summed to 1, generating relative abundances. The normal distribution of α-diversity (Shannon, Simpson, and Chao1) indices and relative taxon abundances (>0.1% abundance of all reads) were assessed using the Shapiro–Wilk test in R software(version 2025.05.0+496) . The α-diversity expressed as the means and the relative abundances of bacteria as least-squares means ± standard error of the mean (SEM) in different datasets were statistically analyzed using analysis of variance (ANOVA) with a mixed model using the emmeans package in R (version 2025.05.0+496), and significant differences were compared using Tukey’s HSD test. The PBM formula and day were fixed effects, and each sample was considered a random effect. A significant difference was considered at *p* ≤ 0.05, and the trend was 0.05 < *p* < 0.10 [23].

## 3. Results

### 3.1. Nutrient Proximate Composition

**Figure 1:**
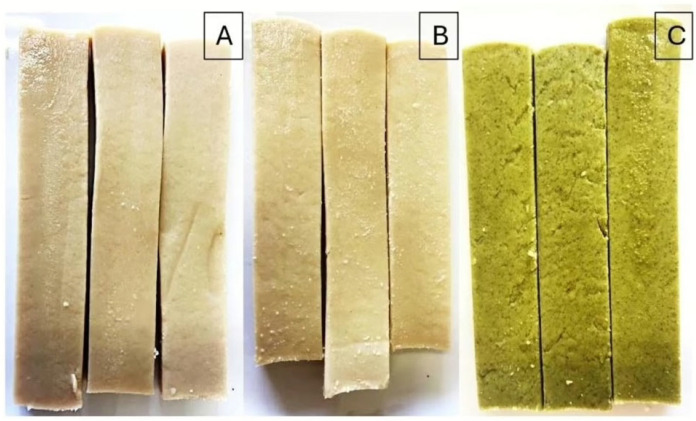
Characteristics of high-moisture plant-based meat in (**A**) 20MB-PBM, (**B**) 30MB-PBM, and (**C**) DW-PBM.

The Sf replacement with Mb Ps and Dw affected the nutrient composition, especially the protein and fat content, compared to the control formula (20MB-PBM) (Table 1; Figure 1). All products from each formula had moisture content higher than 50%, which was categorized as high-moisture PBM [24]. The 30MB-PBM had higher moisture and fat contents, whereas the protein, ash, total carbohydrate, and total energy contents were lower than those of the 20MB-PBM. The DW-PBM had higher fat, total carbohydrates, and total energy, while moisture, protein, and ash content were lower than those of the 20MB-PBM.

### 3.2. Bacterial Diversity

During 28 days of storage, the diversity indices (e.g., Chao1, Simpson, Shannon) of the three PBM formulas varied inconsistently, with 30MB-PBM and DW-PBM having higher diversity indices than 20MB-PBM. In the 1st batch, the Simpson indices of the 20MB-PBM formula were significantly different from those of the five ingredients (*p* ≤ 0.05) (Table 2). Simpson and Shannon indices were significantly different within the group of five ingredients (*p* ≤ 0.05). The alpha diversity of DW-PMB showed a significant difference in Simpson indices (*p* ≤ 0.05), whereas no significant difference in the alpha diversity was observed in the 30MB-PBM during 28 days of storage. In the 2nd batch, the analysis of alpha diversity among the ingredients showed significant differences in the Chao1 index (*p* ≤ 0.05). The Simpson and Shannon indices of the 20MB-PBM and 30MB-PBM formulas were significantly different from those of the other ingredients in the Tukey HSD test (*p* ≤ 0.05).

Beta diversity was calculated using unweighted UniFrac analysis, and distances were estimated among samples from the group of five ingredients and the group of each PBM formula. The three-dimensional scatterplot generated using principal coordinate analysis (PCoA) separated the five ingredients (Figure S1A) for the unweighted analysis. The PCoA plot showed that the bacterial communities in the 20MB-PBM (Figure S1B) were separated during the 5-time sampling points from day 1 to day 28, whereas the bacterial communities in the 30MB-PBM were grouped on days 1 and 21, and the bacterial communities in the DW-PMB were grouped on days 1 and 28 (Figure S1C,D). In the 2nd batch, the PCoA plot separated the five ingredients (Figure S1E). The PCoA plot showed that the 20MB-PBM and 30MB-PBM bacterial communities were separated during the 5-time sampling points from day 1 to day 28 (Figure S1F,G). Only DW-PBM (Figure S1H) was grouped on days 21 and 28.

**Figure 2:**
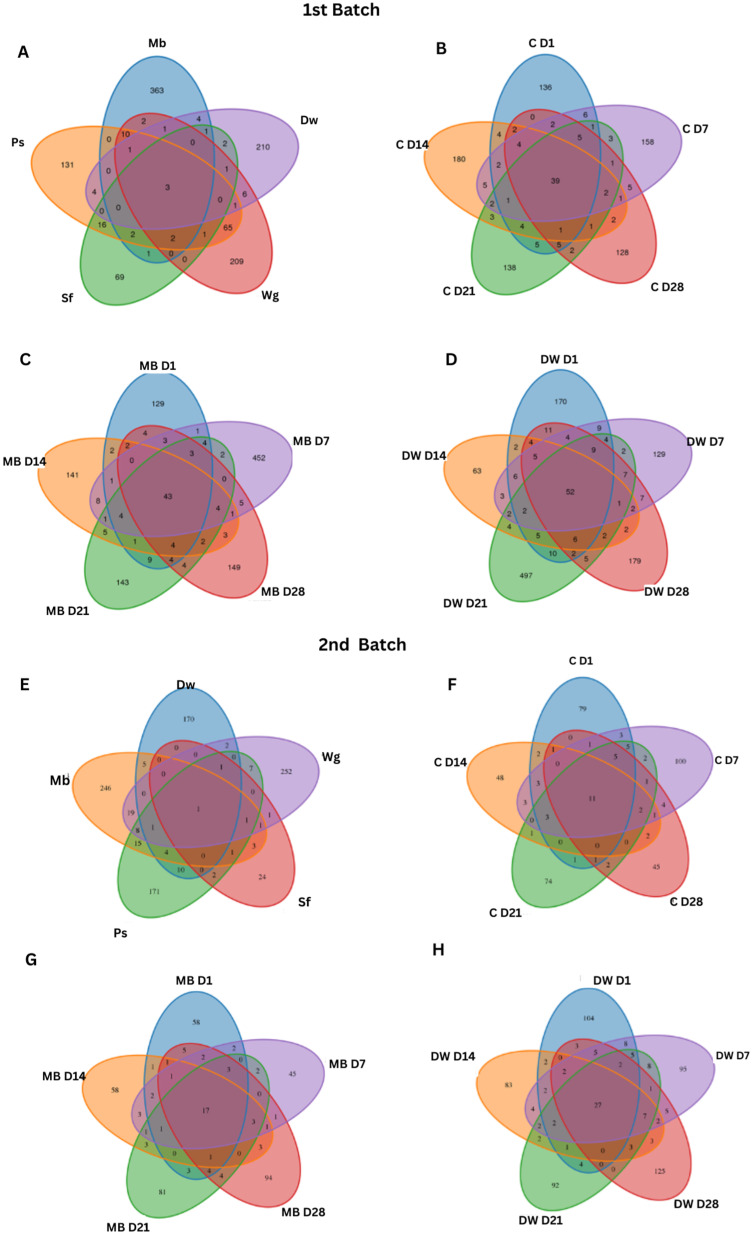
Venn diagram of the bacterial communities in the five ingredients and the high-moisture plant-based meat (PBM) in the control, mung bean protein, and duckweed formula from day 1 (D1) to day 28 (D28). (**A**,**E**) Shared communities in ingredient samples (i.e., Sf: Defated Soy Flour, Ps: Potato Starch, Wg: Wheat Gluten, Mb: Mung bean protein, Dw: Duckweed); (**B**,**F**) shared communities in a control sample (C = 20MB-PBM); (**C**,**G**) shared communities in mung bean protein sample (MB = 30MB-PBM); (**D**,**H**) shared communities in duckweed sample (DW-PBM). (**A**–**D**) represents Batch 1. (**E**–**H**) represents Batch 2.

The Venn diagram provided insights into the core and unique bacterial communities associated with the different ingredients and storage durations of plant-based meat (PBM). The overlap of bacterial communities among the five ingredients was identified, and three counts of common features were identified among the ingredients (Figure 2A). Among the different days of PBM, the 52 common microbes in DW-PBM are visualized using a Venn diagram (Figure 2D). The counts of common features in the 30MB-PMB were 43 communities (Figure 2C), followed by the common microbes in the 20MB-PBM with 39 communities (Figure 2B). In the 2nd batch, only one count of common features among the five ingredients was identified (Figure 2E). Among the different days of PBM, the 27 common microbes in DW-PBM were visualized using a Venn diagram (Figure 2H), and the counts of common features in 30MB-PBM were 17 communities (Figure 2G). The common microbes in the 20MB-PBM with 11 communities were also visualized (Figure 2F).

### 3.3. Bacterial Microbiome of High Moisture Plant-Based Meat (PBM) and the 5 Ingredient Compositions During a Storage Period of 28 Days

**Figure 3:**
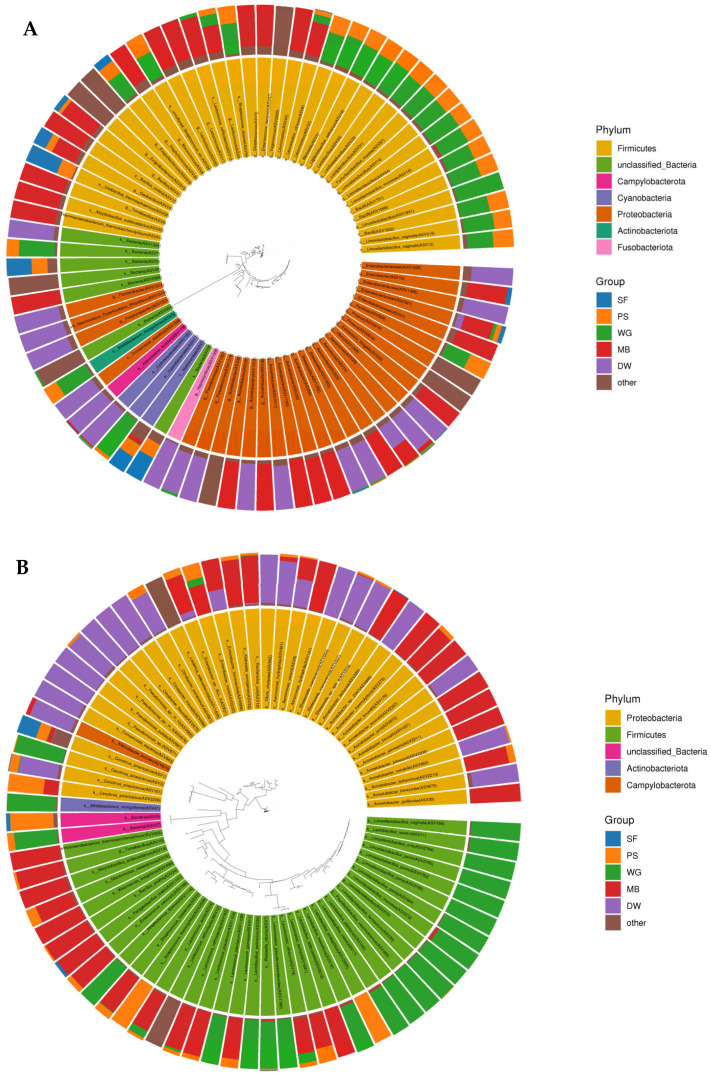
Phylogenetic tree of the bacterial microbiome at the phylum level in the ingredient compositions. (**A**) Batch 1; (**B**) Batch 2. Sf: Defated soy flour, Ps: Potato starch, Wg: Wheat gluten, Mb: Mungbean, Dw: Duckweed, PBM: The groups of three PBM formulas.

The microbial communities in each ingredient composition of the PBM and the 16S rRNA gene sequences were classified into seven bacterial phyla. In the 1st batch (Figure 3A), *Firmicutes* and *Proteobacteria* were the predominant phyla present. *Firmicutes* were abundant in Ps, Wg, and Mb, whereas *Proteobacteria* were abundant at 22% in Mb and 28% in Dw. In the 2nd batch (Figure 3B), *Firmicutes* were abundant in the Wg and Mb, whereas *Proteobacteria* were as highly abundant in Mb and Dw as in the 1st batch.

**Figure 4:**
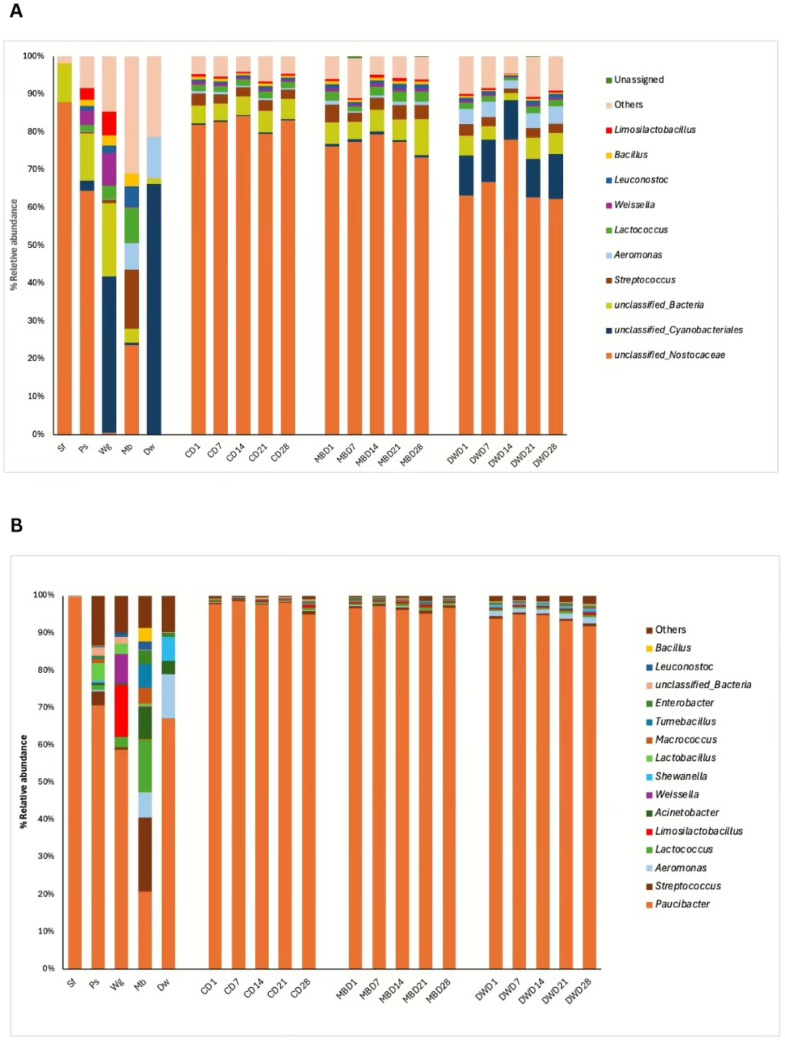
% Relative abundance of the most 10 bacterial communities in ingredient samples (i.e., Sf, Ps, Wg, Mb, Dw) and the high moisture plant-based meat (PBM) in the control (C = 20MB-PBM), mung bean protein (MB = 30MB-PBM, and duckweed (DW = DW-PBM) formula during storage from day 1 (D1) to day 28 (D28) at the genus level (**A**), Batch 1; (**B**), Batch 2.

Based on the relative abundances of the bacterial communities in all samples, the 16S rRNA gene sequences were classified into 743 genera. In the 1st batch (Figure 4A; Table S2), Sf and Ps were dominated by Unclassified_*Nostocaceae*. Unclassified_*Cyanobacteriales* dominated Wg and Dw. Mb showed greater diversity in bacterial communities, such as Unclassified_*Nostocaceae*, *Streptococcus*, *Aeromonas*, and *Lactococcus*. In the 2nd batch, the four ingredients were dominated by *Paucibacter* (>50%), whereas *Paucibacter*, *Streptococcus*, *Aeromonas*, and *Lactococcus* dominated Mb.

For the change in bacterial communities in all PBM samples during storage, the microbial communities of the three PBM formulas were similar during refrigerated storage in the 1st batch (Figure 4A). The relative abundances of *Lactococcus*, *Weissella*, *Leuconostoc*, *Ligilactobacillus, Enterobacter*, and *Geobacillus* significantly differed among the PBM formulas in the 1st batch (*p* ≤ 0.05; Table S2). The relative abundances of *Streptococcus, Leuconostoc*, and *Bacillus* significantly differed by the day of the storage (*p* ≤ 0.05; Table S2). From day 1 to day 28, the levels of Unclassified_*Nostocaceae* (74.6%), Unclassified_*Cyanobacteriales* (4.3%), Unclassified_Bacteria (5.27%), *Streptococcus* (2.8%), and *Lactococcus* (1.7%) in refrigerated PBM changed slightly during storage, which comprised the top five genera of the average relative abundance in all PBM samples (Figure 4A). The unclassified_*Cyanobacteriales* in DW-PBM were more abundant than those in the 20MB-PBM and 30MB-PBM formulas. In the 2nd batch (Figure 4B), the three PBM formulas were significantly different (*p* < 0.01; Table S3). The relative abundances of *Paucibacter* were 97.4% in the 20MB-PBM, 96.4% in the 30MB-PBM, and 93.7% in the DW-PBM groups. From day 1 to day 28, *Paucibacter* (87.8%), *Streptococcus* (1.6%), *Aeromonas* (1.3%), *Lactococcus* (1.3%), and *Limosilactobacillus* (0.9%) comprised the top five genera of the average relative abundance in all PBM samples (Figure 4B).

### 3.4. Relationship Between Change in Bacterial Population and Shelf Life of High Moisture Plant-Based Meat (PBM) of Control, Mung Bean Protein, and Duckweed Formulas During a Storage Period of 28 Days

**Figure 5:**
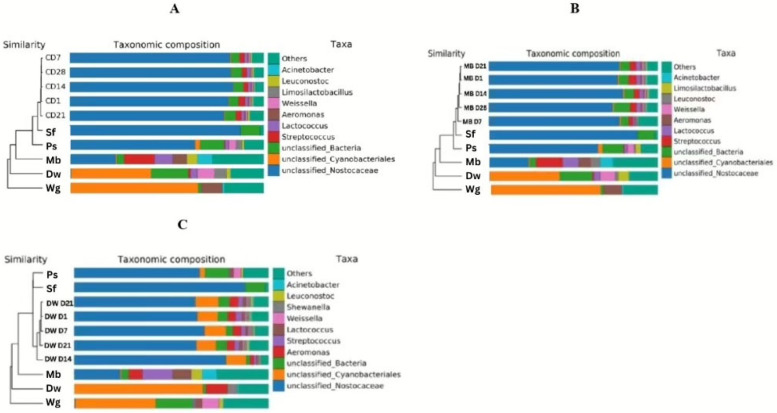

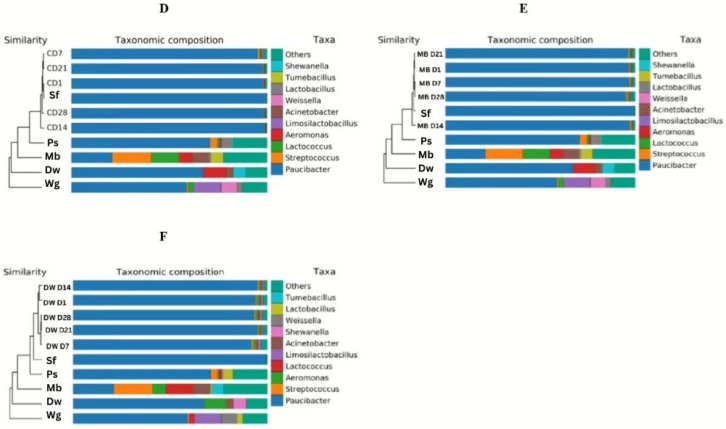
Similarity between the ingredients and the high-moisture plant-based meat (PBM) in the control, mung bean protein, and duckweed formula from day 1 (D1) to day 28 (D28) (method = ClusterTree_bar). The figure legend at the bottom left represents the color of each group in which the cluster tree samples were grouped. The legend in the top right represents the top 10 species abundance in the communities in (**A**,**D**) control formula (C = 20MB-PBM); (**B**,**E**) mung bean protein formula (MB = 30MB-PBM); (**C**,**F**) duckweed formula (DW-PBM), clustered with the ingredient samples (i.e., Sf, Ps, Wg, Mb, Dw). The remaining were classified as others. Unannotated species were classified as unclassified. (**A**–**C**) represents Batch 1. (**D**–**F**) represents Batch 2.

A taxonomic composition histogram with samples closer (with shorter branch lengths) was constructed to analyze and compare the similarity in taxonomic compositions (Figure 5). Sample hierarchical clustering trees of the top 10 genera were constructed to visualize the similarity in genera abundance in all PBM samples. The bacterial compositions of all PBM formulas were closely clustered with the communities in Sf due to the high abundance of Unclassified_*Nostocaceae* and *Paucibacter* but were separately clustered with the communities of other ingredients, especially in Wg and Dw, which had dominant Unclassified_*Cyanobacteriales*. For DW-PBM, the taxonomic composition identified from day 1 to day 28 showed high similarity in abundance, which was also closely clustered with Sf and Ps in the 1st batch (Figure 5C). *Streptococcus, Aeromonas, Lactococcus,* and *Limosilactobacillus* also slightly influenced clustering.

### 3.5. OTU Predictions

**Figure 6:**
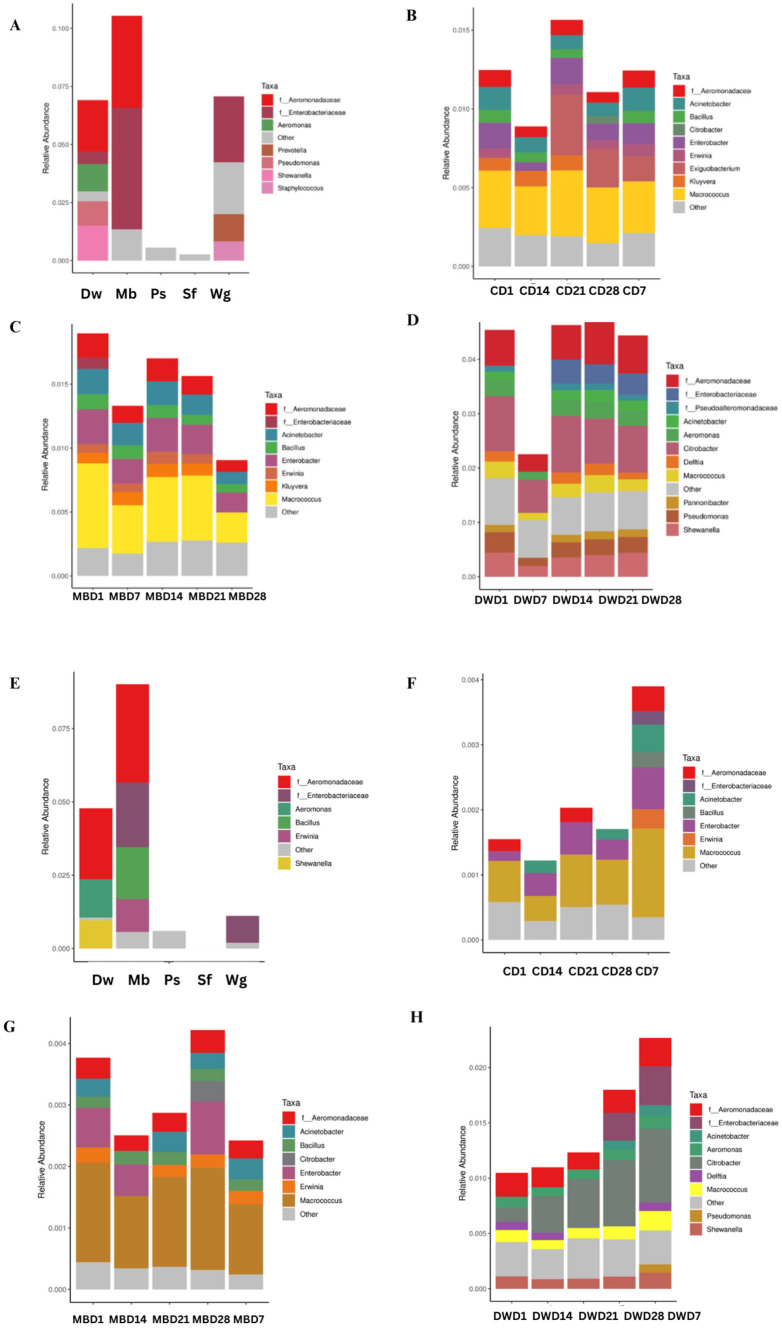
Prediction of BugBase phenotype for potentially pathogenic phenotypes in five ingredients and high-moisture plant-based meat (PBM) in the control, mung bean protein, and duckweed formula from day 1 (D1) to day 28 (D28). (**A**,**E**) Prediction of phenotype in ingredient samples (i.e., Sf, Ps, Wg, Mb, Dw); (**B**,**F**) prediction of phenotype in a control formula (C = 20MB-PBM); (**C**,**G**) prediction of phenotype in mung bean protein formula (MB = 30MB-PBM); (**D**,**H**) prediction of phenotype in duckweed formula (DW-PBM). (**A**–**D**) represents Batch 1. (**E**–**H**) represents Batch 2.

BugBase normalized OTU by predicting the 16S copy number to provide biologically relevant microbiome phenotype predictions at the genus level with the relative abundance of traits estimated over the full range of coverage thresholds (0 to 1) (Figure 6). This study predicted potentially pathogenic phenotypes in five ingredients, and the PBM of control, mung bean protein, and duckweed formulas using BugBase, and their relative abundances were compared. Among the five ingredients in the 1st batch (Figure 6A,E), high phenotype predictions of Potential_Pathogens in Mb were associated with the abundance of *Aeromonadaceae* (0.03) and *Enterobacteriaceae* (0.05). Similarly, Potential_Pathogens prediction was highest due to the abundance of *Aeromonadaceae* (0.2) in Dw, whereas Wg was highest due to the abundance of *Enterobacteriaceae*. The Potential_Pathogens prediction in Ps and Sf was considerably lower. The 20MB-PBM and 30MB-PBM from day 1 to day 28 had similar Potential_Pathogens prediction; the prediction might be related to the similar abundances of *Macrococcus, Exiguobacterium, Enterobacter,* and *Acinetobacter*, while on day 7 of storage, the Potential_Pathogens in Ps and Sf were considerably lower (Figure 6B,C). Similar to DW-PBM, the Potential_Pathogens prediction was lower than that on other days, which might be related to the abundance of *Citrobacter* and *Aeromonadaceae* (Figure 6D).

In the 2nd batch, high phenotype predictions of Potential_Pathogens in Mb and Dw might be related to the abundance of *Aeromonadaceae* (>0.03) (Figure 6F,G). The Potential_Pathogens prediction in Mb (0.03) and Wg (0.01) was the highest due to the abundance of *Enterobacteriaceae*. From day 1 to day 28, the 20MB-PBM and 30MB-PBM had similar Potential_Pathogens prediction, which might be related to the similar abundances of *Macrococcus* and *Enterobacter*. The Potential_Pathogens prediction in DW-PBM was low on day 1 and increased on day 28, which may be related to the abundance of *Citrobacter, Aeromonadaceae,* and *Enterobacteriaceae* (Figure 6H).

## 4. Discussion

Despite this vulnerability, emerging high-moisture extrusion technology is being proposed as an alternative, and the creation of appealing alternative meats has a limited shelf life. The addition of plant proteins (e.g., soy, wheat, and bean) provides structural properties similar to those of animal-based meat [24]; however, the PBM poses a more significant risk of microbial contamination than other foods. Because of their high moisture and protein content, they are ideal for the growth of microorganisms. The scientific literature on the bacterial microbiome of plant proteins is limited. The present study applied NGS-based 16S rRNA gene sequencing, allowing us to uncover the major bacterial populations of bacterial communities, including unculturable bacteria, in the five raw ingredients and the PBM containing 30% mung bean protein or 3% duckweed formulas. 16S rRNA sequencing identified potential sources of heat-stable pathogens from raw ingredients that survive under the extruder. In contrast, the conventional method could not detect unculturable microbes on agar plates, such as *Nostocaceae* and *Paucibacter* in Sf and *Cyanobacteriale* in Wg and Dw ingredients.

The various protein sources of the ingredients showed different microbial attributes, which led to microbiome diversity (both alpha and beta diversity) in PBM. Protein sources have different attributes that influence their specific chemical properties and bacterial communities [2]. *Firmicutes* and *Proteobacteria* were the major bacterial phyla in the five raw ingredients. Our results showed that *Firmicutes* dominated potato starch, wheat gluten, and mung bean, while *Proteobacteria* dominated mung beans and duckweed. *Proteobacteria* pose several pathogenic risks, indicating that mung beans and duckweed can contain microbial pathogens. This was confirmed by a recent report on contaminated duckweed, which can result from the source of water [6]. Storage time and production batch significantly influenced the bacterial community of PBM. The presence of Unclassified_*Nostocaceae* and Unclassified_*Cyanobacteriale* in the 1st batch and *Paucibacter Streptococcus*, *Aeromonas*, *Lactococcus*, and *Limosilactobacillus* in the 2nd batch played essential roles in the bacterial profiles of PBM during storage. Shelf-life prediction based on the taxonomic composition from day 1 to day 28 indicated that adding 30% mung bean protein and 3% duckweed had similar taxonomic compositions, indicating that PBM containing 30% mung bean protein and 3% duckweed was still safe. There was no change in the appearance of the 30MB-PBM and DW-PBM products, although greater microbial diversity was observed in the 30MB-PBM and DW-PBM.

Similar to the Venn diagram, only three bacterial communities were shared among the five ingredients, indicating the presence of certain core microflora in each ingredient source. The bacterial compositions in the raw ingredients of mung bean protein and duckweed were more diverse, reflecting greater diversity in 30MB-PBM and DW-PBM. Accordingly, the microbial load of raw ingredients strongly influenced the bacterial profiles of PBM. This can give rise to issues impacting the safety and quality of ingredient sources and the final bacterial communities in the finished products [24]. Differences in the nutritional profile, pH, and texture of the ingredients may influence the survival and growth of both spoilage and pathogenic microorganisms [25,26]. For instance, the clustering of bacterial compositions in the PBM was closely clustered with the communities in Sf in both Batch 1 and 2, indicating that defatted soy flour was the primary influencer of the bacterial profiles in all PBM.

After texturization in a twin-screw extruder at 130 °C, the process, which has a mechanical shear force, heat, and pressure, causes considerable changes in the molecular structures of the protein and bacterial communities [27]. Based on the diversity and bacterial profiles, heat-stable bacteria (*Cyanobacteria*) or spore-forming bacteria (*Bacillus*) in the PBM, originating from the raw ingredients, were still observed after extrusion, indicating that the quality of the raw ingredients needs to be checked. This indicates that microbial growth occurs at all stages of PBM production, including in the finished products [28]. Since DNA was extracted from the ingredient powder, some bacteria in the bacterial microbiome of several ingredients were still observed after extrusion. Although thermal processing stages are included in their production process, PBMs with near-neutral pH levels and high protein and moisture concentrations are more susceptible to rapid spoilage [29]. Pathogen contamination can be transferred from raw materials during handling, cooking extrusion, packing [28], or via environmental contamination. For instance, Unclassified _*Nostocaceae* and *Paucibacter* in SF may originate from the water used to clean soybeans before soybean flour production [30]. Prakash et al. [31] reported that wheat flour is a potential source of the enteric pathogen, according to our result’s wheat gluten microbiome.

16S rRNA gene sequencing allowed the identification of unexpected *Cyanobacteria* in Batch 1, whereby *Paucibacter* was the majority in the ingredients of Batch 2. A possible explanation for this is the competition between bacterial groups (*Paucibacter* and *Cyanobacteria*) [30,32]. This resulted in a significant change in the bacterial microbiome of the ingredients during storage. Available data confirm that *Sphingomonas, Pseudomonas, Sphingosinicella, Sphingopyxis, Paucibacter,* and *Burkholderia* have been reported to degrade *cyanobacteria* [29]. In addition to our results, *Lactococcus, Weissella, Leuconostoc, Ligilactobacillus, Enterobacter*, and *Geobacillus* were dominant in batch 1, and *Streptococcus, Leuconostoc,* and *Bacillus* changed significantly during storage, which reflected the predominance of *Paucibacter* in Batch 2. The degradation of *cyanobacteria* in our study may be confirmed by Kormas and Lymperopoulou [32], who revealed that the bacterial genera *Arthrobacter, Bacillus,* and *Lactobacillus* play a significant role in degrading cyanobacteria and their toxins.

Ensuring food safety and prolonging the shelf life of PBM is crucial throughout its production chain. High-throughput sequencing technology, such as 16S rRNA sequencing, has been used in microbiome studies to determine the bacteria that caused the spoilage of the PBM. The predicted 16S copy number to BugBase phenotype for potentially pathogenic phenotypes in five ingredients and plant-based meat (PBM) of control, mung bean protein, and duckweed formulas. We found a relationship between the Potential_Pathogens in Mb, Dw, and DW-PBM, which was related to the abundance of *Aeromonadaceae.* Wg was associated with the abundance of *Enterobacteriaceae*. The prediction indicated the predominant spoilage bacterial flora, which played a role in the shelf life and pathogenic risk. The presence of *Enterobacteriaceae* in heat-treated PBM suggests that post-process contamination occurred, and *Enterobacteriaceae* proliferation in refrigerated samples has been implicated [33]. Some endospore-forming bacteria (e.g., *Clostridium spp*. or *Bacillus spp*.) and other bacteria (e.g., *Lactobacillus sakei* and *Enterococcus faecium*) are the major cause of spoilage that can survive the heating under the extrusion process, whereby re-contamination can occur in the final product [34]. The results of the present study showed similar bacterial communities in PBM. From day 1 to day 28, the Potential_Pathogens prediction in DW-PBM was related to *Citrobacter*, *Aeromonadaceae*, and *Enterobacteriaceae*. Our results indicated that PBM products require sufficient and adequate thermal processing before consumption, as some researchers observed the growth of *Enterobacteriaceae* to be slightly higher than that of animal-based meat [34].

Another study reported that plant-based meat spoilage and pathogenic microorganisms proliferated faster in PBM at abused storage temperatures (22 °C and 32 °C) [33]. The influence of microbial contamination on the safety of PBM is another issue with PBM products after hot meal preparation. Accordingly, PBM requires appropriate storage conditions to preserve its high-quality standards [33] and prevent environmental contamination during storage. Storage at a cool temperature (4 °C) has been reported to cause significant loss associated with open-air storage and decreased shelf life over 10 consecutive days [24,35]. Our results showed that the bacterial communities in the three PBM formulas did not significantly change at different storage times. Based on the profiles of 30MB-PBM and DW-PBM, 28 days of storage did not cause a considerable shift in the bacterial community. In the present study, cold storage extended the shelf life of PBM containing mung bean protein and duckweed, which is similar to the findings of Hai et al. [27]. Duckweeds contain beneficial secondary metabolites like phenolic compounds, with OH groups essential for their antimicrobial and antioxidant properties. Accordingly, adding polyphenolic-rich plants to meat has been reported to improve the safety and quality of foods [36]. However, *Enterobacteriaceae* and *Aeromonadaceae* need to be monitored during longer cold storage of PBM, as they predict potentially pathogenic phenotypes.

## 5. Conclusions

The application of alternative plant-based proteins raises questions regarding food safety hazards and the presence of microbial species in PBM products. The PBM containing 30% mung bean protein and 3% duckweed did not change the appearance of the 30MB-PBM and DW-PBM products for 28 days of storage at 2–4 °C in a refrigeration room. 16S rRNA gene sequencing allowed us to identify unexpected unculturable microbes, such as *Nostocaceae in* the soybean flour and *Cyanobacteriale* in the mung bean and duckweed in the 1st batch. Apart from the effect of batches, *Paucibacter*, degrading cyanobacteria, was the majority ingredient population, indicating a change in the bacterial population in the soybean flour during storage at room temperature in the 2nd batch. Therefore, the ingredient composition was the key factor influencing the microbiome of the final PBM product. Our research on food safety and quality concerns the persistence of pathogenic bacteria and microbial contamination in the final products, with a specific focus on the survivability of spore-forming bacteria (*Bacillus*) and possible pathogens (*Enterobacteriaceae*) after thermal processing and extrusion. Implementing food safety management throughout the production chain is essential to ensure that high-moisture plant-based meat is of high quality and safe for human consumption. This includes the careful selection of raw materials, optimized extrusion parameters, and proper post-processing handling and packaging. However, this study was limited to a specific storage condition at 2–4 °C and may differ under different conditions. The integrated results of 16S rRNA gene sequencing with the culturing method or qPCR for the detection of potential pathogens will be useful for risk assessment. Further research on post-packaging microbial transformations will bridge the gap between the identification of microbial risks and the implementation of control strategies for PBM products. Furthermore, more attention must be paid to the recommended storage conditions, such as temperature control, safe packaging methods, and proper hygiene habits.

## Figures and Tables

**Table 1:** Nutrient proximate compositions of the high-moisture plant-based meat (PBM) in the control, mung bean protein, and duckweed formula.

Items	20MB-PBM	30MB-PBM	DW-PBM
Moisture(%)	62.54	68.40	61.49
Protein (%)	26.91	21.38	25.75
Fat (%)	1.36	2.86	3.50
Ash (%)	1.77	1.16	1.55
Total carbohydrate(%)	7.42	6.20	7.71
Total energy (Kcal/100g)	149.56	136.06	165.34

**Table 2:** Alpha diversity of bacterial populations in the ingredients and high-moisture plant-based meat (PBM) in the control, mung bean protein, and duckweed formulas.

Samples	Diversity Indices (Mean ± SE)			*p*-Value ^1^		
	Chao1	Simpson	Shannon	Chao1	Simpson	Shannon
1st batch						
Individual Ingredients	252 ± 54.6 ^a^	0.61 ± 0.06 ^a^	2.87 ± 0.35 ^a^	0.719	0.005	0.015
20MB-PBM	224 ± 54.6 ^a^	0.32 ± 0.06 ^b^	1.53 ± 0.35 ^a^	<0.01	<0.01	<0.01
30MB-PBM	287 ± 54.6 ^a^	0.41 ± 0.06 ^ab^	1.95 ± 0.35 ^a^	0.4309	0.349	0.408
DW-PBM	323 ± 54.6 ^a^	0.54 ± 0.06 ^ab^	2.49 ± 0.35 ^a^	0.217	0.026	0.067
2nd batch						
Individual Ingredients	210 ± 25.8 ^a^	0.561 ± 0.07 ^a^	2.74 ± 0.38 ^a^	0.011	<0.01	<0.01
20MB-PBM	105 ± 25.8 ^b^	0.058 ± 0.07 ^b^	0.34 ± 0.38 ^b^	<0.01	0.45	0.382
30MB-PBM	110 ± 25.8 ^ab^	0.086 ± 0.07 ^b^	0.47 ± 0.38 ^b^	0.893	0.798	0.813
DW-PBM	166 ± 25.8 ^ab^	0.346 ± 0.07 ^ab^	1.29 ± 0.38 ^ab^	0.113	<0.01	0.094

^1^ Difference within the group of ingredients or each formula group of PBM in each batch, stored from day 1 to day 28. Superscript letters indicate a significant difference in the diversity index in the column (ANOVA and Tukey HSD test, *p* ≤ 0.05) for each batch.

## Data Availability

Raw sequences were deposited in the NCBI BioProject database under accession number PRJNA1259754.

## Supplementary Materials

The following supporting information can be downloaded at: https://www.mdpi.com/article/10.3390/biology14060735/s1, Figure S1: Principal coordinate analysis (PCoA) plot with Bray-Curtis dissimilarity of the bacterial population in the high moisture plant-based meat (PBM) in control, mung bean protein, and duckweed formula. (A–D) represent replicate 1. (E–H) represent replicate 2. (A,E) Bray-Curtis PCoA ordination of ingredient composition samples (i.e., Sf, Ps, Wg, Mb, Dw); (B,F) Ordination of a control sample (C = 20MB-PBM); (C,G) Ordination of mung bean protein sample (MB = 30MB-PBM); (D,H) Ordination of duckweed sample (DW-PBM). Each dot represents one sample. The samples were colored based on the grouping information, if applicable. The confidence ellipse defines a region that contains 95% of all samples that can be drawn from the underlying Gaussian distribution. X-axis: First principal component. The percentage value indicates the contribution of PC1 to variability. Y-axis: Second principal component. The percentage value indicates the contribution of PC2 to variability; Table S1: Composition of high-moisture plant-based meat in (A) control, (B) mung bean protein, and (C) duckweed formula; Table S2: Selected bacterial genera (30 most abundant genera (>0.1 relative abundance), % of all reads) in all samples of high-moisture meat analog in the 1st batch; Table S3: Selected bacterial genera (26 most abundant genera (>0.1 relative abundance), % of all reads) in all samples of high-moisture meat analog in the 2nd batch.
