# Supplementary material for: Exploring the Bacterial Microbiome of High-Moisture Plant-Based Meat Substituted Soybean Flour with Mung Bean Protein and Duckweed Powder
Source: Biology (Basel). 2025 Jun 19;14(6):735. doi: 10.3390/biology14060735 (PMC12189225; doi:10.3390/biology14060735)
Supplement: Supplementary file 1 [file biology-14-00735-s001.zip › biology-3661386-supplementary.pdf]

Supplementary Materials

Table S1. Composition of high moisture plant-based meat in (A) control and (B) mung bean protein, and (C) duckweed formula.

	Ratio (% w/w) in each formula		
**Ingredients**	**C-PBM**	**MB-PBM**	**DW-PBM**
Defatted soy flour	50	30	28.5
Wheat gluten	30	30	30
Mung bean	20	30	28.5
Potato flour	0	5	5
Canola Oil	0	5	5
Duckweed	0	0	3

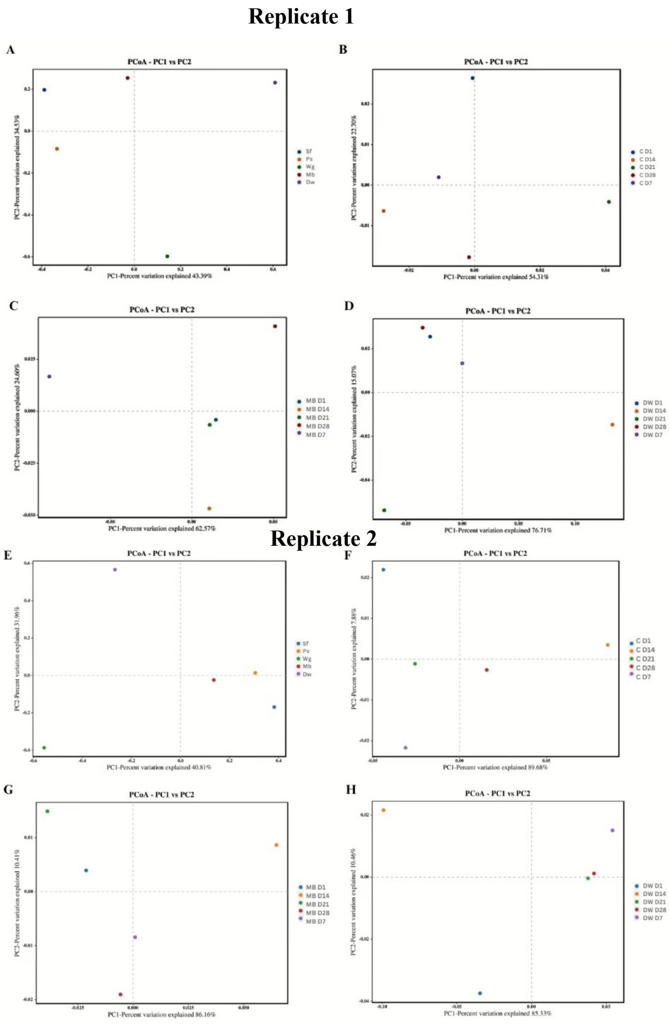

Figure S1. Principal coordinate analysis (PCoA) plot with Bray-Curtis dissimilarity of the bacterial population in the high-moisture plant-based meat (PBM) in the control, mung bean protein, and duckweed formula. Figure A-D represent replicate 1. Figure E-H represent replicate 2. (A,E) Bray-Curtis PCoA ordination of ingredient composition samples (i.e., Sf, Ps, Wg, Mb, Dw); (B,F)

Ordination of a control sample (C=20MB-PBM); (C,G) Ordination of mung bean protein sample(MB=30MB-PBM); (D,H) Ordination of duckweed sample (DW-PBM). Each dot represents one sample. The samples were colored based on the grouping information, if applicable. The confidence ellipse defines a region that contains 95% of all samples that can be drawn from the underlying Gaussian distribution. X-axis: First principal component. The percentage value indicates the contribution of PC1 to variability. Y-axis: Second principal component. The percentage value indicates the contribution of PC2 to variability.

Table S2. Selected bacterial genera (30 most abundant genera (>0.1 relative abundance), % of all reads) in all samples of high moisture meat analog in the 1st batch.

**Genus**	**Sample type**			**SEM**	***p*-value**	
	**Control (20MB-PBM)**	**Mungbean (30MB-PBM)**	**Duckweed (DW-PBM)**		**Formula**	**Day**
Unclassified_*Nostocaceae*	82.3	76.8	66.6	1.480	<0.01	0.105
Unclassified_ *Cyanobacteriales*	0.4	0.7	10.8	0.180	<0.01	0.422
Unclassified _Bacteria	5.0	6.2	4.4	0.542	0.108	0.142
Streptococcus	2.6	3.5	2.3	0.199	<0.01	0.030
Aeromonas	0.5	0.8	3.8	0.254	<0.01	0.362
Lactococcus	1.7	2.2	1.5	0.149	0.019	0.154
Weissella	0.6	0.7	0.5	0.058	0.047	0.346
Leuconostoc	0.6	1.0	0.7	0.064	0.012	0.027
Acinetobacter	0.4	0.6	0.5	0.062	0.207	0.572
Bacillus	0.7	0.8	0.4	0.039	<0.01	0.035
Limosilactobacillus	0.5	0.6	0.4	0.062	0.151	0.752
Macrococcus	0.7	0.9	0.5	0.072	0.010	0.099
Tumebacillus	0.4	0.5	0.4	0.031	<0.01	0.146
Shewanella	0.0	0.0	1.6	0.112	<0.01	0.461
Unclassified_ Enterobacteriaceae	0.2	0.3	0.6	0.014	<0.01	<0.01
Lactobacillus	1.7	2.2	1.5	0.149	<0.01	0.068
Ligilactobacillus	0.2	0.2	0.1	0.016	0.030	0.296
Pseudomonas	0.1	0.1	0.6	0.042	<0.01	0.472
Unclassified_Bacilli	0.2	0.2	0.1	0.016	0.131	0.146
Enterobacter	0.2	0.3	0.2	0.019	0.016	0.090
Exiguobacterium	0.8	0.0	0.1	0.237	0.079	0.404
Lacticaseibacillus	0.1	0.1	0.1	0.015	0.211	0.571
Vibrio	0.0	0.0	0.3	0.018	<0.01	0.461
Proteus	0.0	0.7	0.0	0.393	0.436	0.482
Escherichia_Shigella	0.1	0.2	0.1	0.015	0.010	0.392
Arcobacter	0.0	0.0	0.2	0.017	<0.01	0.541
Bifidobacterium	0.1	0.1	0.0	0.006	<0.01	0.078
Comamonas	0.0	0.0	0.2	0.015	<0.01	0.218
Geobacillus	0.1	0.1	0.1	0.012	0.025	0.805
Enterococcus	0.1	0.1	0.1	0.022	0.070	0.142

Values are least-squares means of relative abundance ± standard error of the mean (SEM).

Table S3. Selected bacterial genera (26 most abundant genera (>0.1 relative abundance), % of all reads) in all samples of high moisture meat analog in the 2nd batch.

Genus	Sample type			SEM	*p*-value	
	Control (20MB-PBM)	Mungbean (30MB-PBM)	Duckweed (DW-PBM)		Formula	Day
Paucibacter	97.400	96.400	93.700	0.427	<0.01	0.118
Streptococcus	0.431	0.675	0.628	0.066	<0.01	0.147
Aeromonas	0.105	0.165	1.308	0.055	<0.01	0.283
Lactococcus	0.342	0.579	0.520	0.057	<0.01	0.140
Limosilactobacillus	0.231	0.295	0.307	0.036	<0.01	0.151
Acinetobacter	0.100	0.143	0.225	0.020	<0.01	0.057
Weissella	0.142	0.141	0.148	0.022	<0.01	0.148
Shewanella	0.000	0.000	0.499	0.026	<0.01	0.454
Lactobacillus	0.048	0.076	0.084	0.009	<0.01	0.459
Macrococcus	0.152	0.276	0.223	0.025	<0.01	0.224
Tumebacillus	0.092	0.148	0.128	0.014	<0.01	0.150
Enterobacter	0.095	0.120	0.218	0.023	<0.01	0.158
Unclassified _Bacteria	0.157	0.127	0.186	0.026	<0.01	0.188
Leuconostoc	0.076	0.107	0.151	0.012	<0.01	0.130
Bacillus	0.073	0.097	0.080	0.010	<0.01	0.381
Citrobacter	0.002	0.005	0.201	0.033	<0.01	0.461
Lacticaseibacillus	0.041	0.032	0.048	0.006	<0.01	0.091
Pseudomonas	0.008	0.009	0.152	0.011	<0.01	0.470
Ligilactobacillus	0.035	0.056	0.042	0.009	<0.01	0.407
Escherichia_Shigella	0.030	0.041	0.037	0.006	<0.01	0.121
Bifidobacterium	0.012	0.018	0.013	0.003	<0.01	0.128
Comamonas	0.003	0.003	0.082	0.004	<0.01	0.626
Vibrio	0.000	0.000	0.100	0.006	<0.01	0.461
Unassigned	0.009	0.000	0.003	0.002	<0.01	0.098
Geobacillus	0.015	0.026	0.028	0.006	<0.01	0.763
Enterococcus	0.018	0.024	0.036	0.007	<0.01	0.159

Values are least-squares means of relative abundance ± standard error of the mean (SEM).
